# Author Correction: Global surveillance of antimicrobial resistance in food animals using priority drugs maps

**DOI:** 10.1038/s41467-024-45494-7

**Published:** 2024-01-31

**Authors:** Cheng Zhao, Yu Wang, Ranya Mulchandani, Thomas P. Van Boeckel

**Affiliations:** 1 Health Geography and Policy Group, ETH Zürich, Zürich, Switzerland; 2 One Health Trust, Washington, DC USA; 3 Spatial Epidemiology Lab, Université Libre de Bruxelles, Brussels, Belgium

**Keywords:** Ecological epidemiology, Antimicrobial resistance, Epidemiology, Geography, Health policy

Correction to: *Nature Communications* 10.1038/s41467-024-45111-7, published online 26 January 2024

In this article the funding from ‘the Swiss National Science Foundation’ was omitted. The original article has been corrected.

