# Supplementary material for: Global surveillance of antimicrobial resistance in food animals using priority drugs maps
Source: Nat Commun. 2024 Jan 26;15:763. doi: 10.1038/s41467-024-45111-7 (PMC10817973; doi:10.1038/s41467-024-45111-7)
Supplement: Supplementary file 1 — Supplementary Information [file 41467_2024_45111_MOESM1_ESM.pdf]

Supplementary Information for

Global surveillance of antimicrobial resistance in food animals using priority drugs maps.

Cheng Zhao[1], Yu Wang[1], Ranya Mulchandani[1], Thomas P. Van Boeckel[1,2,3,*]

*Corresponding author: thomas.van.boeckel@gmail.com

Affiliations:
1 Health Geography and Policy Group, ETH Zürich, Switzerland.
2 One Health Trust, Washington DC, USA.
3 Spatial Epidemiology Lab, Université Libre de Bruxelles, Belgium.

Supplementary Information

Supplementary Methods

Literature review and data extraction

We searched for point-prevalence surveys (PPS) published between 2000 and 2019 reporting antimicrobial resistance in healthy food animals in low- and middle-income countries, focusing on *Escherichia coli* and nontyphoidal *Salmonella* spp.. The literature search was conducted in three rounds from four databases - PubMed, Scopus, ISI Web of Science, and China National Knowledge Infrastructure. The first round was conducted on 28.03.2019 from the first three aforementioned databases, and extracted data from all papers published between January 2000 and December 2018. The extracted data and details of literature review were published in Van Boeckel and Pires *et al.* 2019. The second round of literature search was conducted on 11.03.2020 from all four databases, and included surveys published between January 2000 and December 2019 exclusively for China. The extracted data and details of literature review were published in Zhao *et al.* 2020. The third round of literature search was conducted on 12.01.2022 from the first three aforementioned databases, and included all papers published between January 2019 and December 2019 in low- and middle-income countries apart from China. The search queries used for the third round of literature review was the same as in Van Boeckel and Pires *et al.* 2019. Zotero (version 5.0.96.2) and Microsoft Excel (version 16.53) were used for the literature review.

All three rounds of literature review were conducted with the following procedure (Supplementary Table 1). First, we screened in total 44,325 titles and abstracts, and excluded 40,702 non-PPS publications. We read 3,623 manuscripts in full, and excluded strain surveys, surveys on diseased animals, surveys conducted on a mixture of animal species, surveys without subnational geographic information, and other non-PPS surveys. After the exclusion, there were 1,360 PPS suitable for AMR mapping purposes. We further excluded animal species with small sample sizes such as camel and buffalo, and excluded drug-pathogen combinations not considered in this analysis such as *Campylobacter* and erythromycin. After the exclusion, 1,088 PPS that reported resistance prevalence in *Escherichia coli* and nontyphoidal *Salmonella* spp to 7 antimicrobials (listed in Methods section) were retained for the analyses. All data used in the current analyses are available in the supplementary file, and can also be downloaded at https://resistancebank.org.

Antimicrobial susceptibility testing in the PPS was conducted using either diffusion methods or dilution methods. The majority of PPS used diffusion methods, including disk diffusion (79%) and E-test (0.2%). The rest of PPS used dilution methods, including broth dilution (14%), agar dilution (5%), and automated devices such as VITEK2 (2%). Among the PPS, there was no systematic difference in the measurements between these two families of methods[1]. In each PPS, antimicrobial susceptibility testing results are compared with breakpoints to determine resistance, which are provided by laboratory guidelines and revised annually. Only 18% of records reported the breakpoints used. However, the majority (93%) of PPS mentioned the name of laboratory guidelines used, and 66% among these also mentioned the year of the guideline. The guidelines mentioned by the PPS included guidelines published by the Clinical & Laboratory Standards Institute (96%), the European Committee on Antimicrobial Susceptibility Testing (3%), and the French Society of Microbiology (1%). We adjusted for variations of breakpoints used between surveys, using a

method developed by Van Boeckel and Pires *et al.* 2019 in section "Harmonization of Antimicrobial Resistance Rates" in the Supplementary Material of the reference publication[1]. The adjustment resulted in 635 (2%) resistance prevalence being revised.

Imputation of missing data on resistance prevalence for mapping priority antimicrobials

Missing resistance prevalence data in the point-prevalence surveys were imputed using Multivariate Imputation by Chained Equations (MICE)[2]. Using MICE, a set of plausible values for the missing resistance prevalence could be inferred from the distribution of reported resistance prevalence data, using specified imputation models. The prediction accuracy of three imputation models were compared: Bayesian linear regression (BLR), LASSO regression (LASSO-GLM), and feed-forward neural network (NN). For NN, we selected the optimal combination of hyperparameters, by comparing the root-mean-square error (RMSE) of the imputed values created using NN models with 500 different hyperparameters. These hyperparameters were drawn randomly from the following ranges: the number of nodes of the hidden layer between 1 to 272, dropout rate between 0.2 and 0.8, and learning rate between 0.00001 and 0.1. The lowest value of RMSE was generated with 145 nodes on the hidden layer, a dropout rate of 0.4, and a learning rate of 0.0001.

The comparison of imputation methods was conducted as following. First, we selected a subset of 272 surveys, which contained no missing values of resistance prevalence for the 7 antimicrobials listed in the Methods section. Second, we conducted 50 Monto Carlo simulations to estimate the accuracy of each imputation method. Concretely, for each simulation, we randomly removed 2 out of 7 reported antimicrobial resistance prevalence in each survey, and conducted 4-fold spatial cross validation to impute these deleted values back. These 4 spatial folds were determined based on the continents of the survey locations: America, Africa, western Asia, and eastern Asia. Finally, we compared the RMSE of the imputed missing values of each fold for all Monto Carlo simulations, by running MICE with different imputation methods. The prediction accuracy of LASSO-GLM (RMSE 26.6) outperformed BLR (RMSE 28.7) and NN (RMSE 27.0). Additionally, adding an ad-hoc step of predictive mean matching, and including additional covariates in the imputation process did not improve the prediction accuracy.

We conducted imputation on a subset of 806 PPS that reported at least 4 out of the 7 drugs. These PPS contained 1,411 resistance profiles – some PPS reported resistance profiles for multiple animal species or for multiple sample types. Using MICE combined with LASSO-GLM, we imputed 2,117 (21%) missing values out of 9,877 resistance prevalence. The number of imputed resistance prevalence was 720 for cefotaxime (51%), 375 (27%) for sulfamethoxazole-trimethoprim, 306 (22%) for chloramphenicol, 202 (14%) for ampicillin, 196 (14%) for tetracycline, 195 (14%) for ciprofloxacin, and 123 (9%) for gentamicin. We conducted 10 multiple imputations, each with 25 iterations.

Mapping resistance prevalence for each antimicrobial

We mapped the prevalence of resistance for each antimicrobial using Gaussian process stacked generalization[3]. The mapping procedure included two steps. In the first step, we trained three 'child models' to predict resistance prevalence based a set of environmental and anthropogenic covariates (Supplementary Table 3). For each antimicrobial, we also included its estimated amount of use divided by the estimated biomass of food animals in 2020[4] as a

covariate in the corresponding child models. The child models included boosted regression trees[5] (BRT), least absolute shrinkage and selection operator applied to linear regression[6] (LASSO-GLM), and feed-forward neural network implemented in Keras[7] (FFNN). The models were trained using four-fold spatial-cross validation (Supplementary Figure 15). For the BRT model, we applied a tree complexity of 3 with 50 initial trees, a learning rate of 0.0005, and a step size of 50. For the NN model, we applied one hidden layer with 31 nodes, a dropout rate of 0.49 and a learning rate of 0.01, using adaptive moment estimation optimizer, and the rectified linear activation function for each layer.

In the second step, the child model predictions were stacked using Gaussian process regression, fitted using the integrated nested Laplace approximations (INLA)[8]. This second step allowed to simultaneously capture the influence of environmental and anthropogenic covariates, as well as the residual spatial correlation. INLA is a deterministic method for Bayesian inference in latent Gaussian modelling, and is comparatively faster than other inference methods such as Markov chain Monte Carlo. The INLA formula included the child model predictions of resistance prevalence as fixed effects, and the spatial autocorrelation as a random effect. The coefficients of the fixed effects were constrained between 0 and 1, such that the coefficients approximately sum to one[3]. The residual spatial correlation was modelled as a Gaussian Markov random field (GMRF) with a Matern covariance function. The prior of the range for the covariance function was set at 4.06 decimal degrees, or roughly 487 km at equator, based on previous work on spatial correlation of AMR[9]. We constructed the mesh – on which the GMRF representation was built – using a cutoff of 0.005 decimal degrees, a maximum edge of 1 and 4 decimal degrees, an offset of 0.25 and 1.5 decimal degrees, for the inner domain and outer extension respectively.

Supplementary Figures

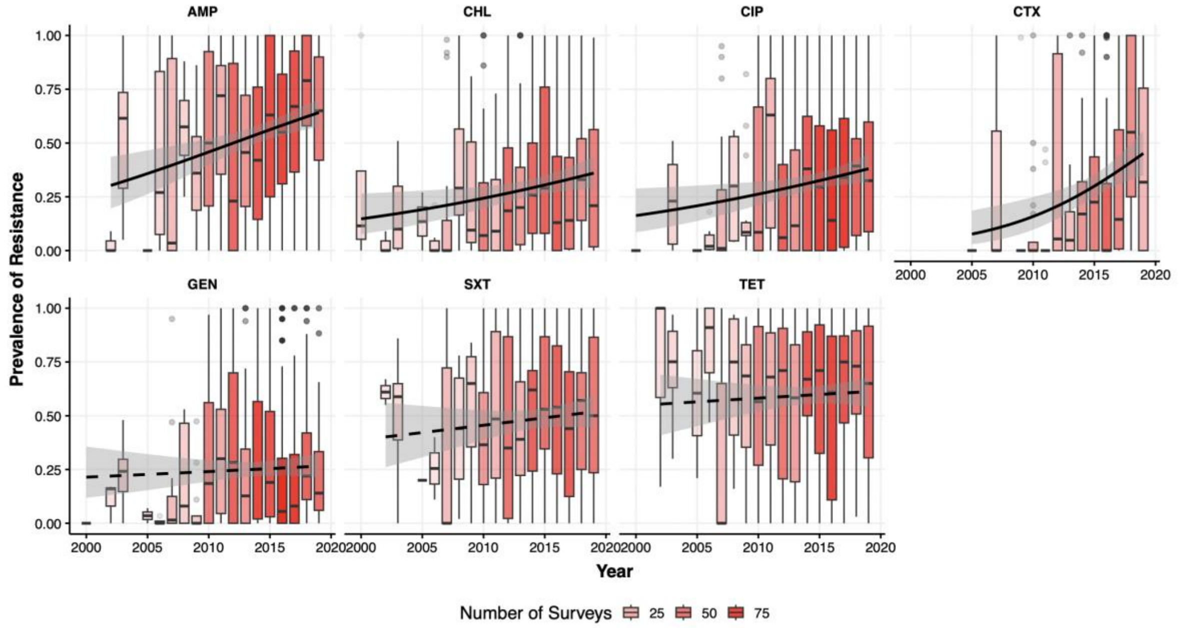

Supplementary Figure 1. **Chicken**: temporal trends of the prevalence of resistance, for ampicillin (AMP, n = 588, p = 0.00038), chloramphenicol (CHL, n = 516, p = 0.017), ciprofloxacin (CIP, n = 624, p = 0.015), cefotaxime (CTX, n = 316, p = 0.00069), gentamicin (GEN, n = 647, p = 0.55), sulfamethoxazole-trimethoprim (SXT, n = 486, p = 0.28), and tetracycline (TET, n = 567, p = 0.55). Solid lines represent significant temporal trends ($p < 0.05$), and dashed lines represent nonsignificant trends. Transparency levels of the red colors were proportional to the number of surveys published each year. Temporal trends were significant (p value < 0.05) for AMP, CHL, CIP, and CTX.

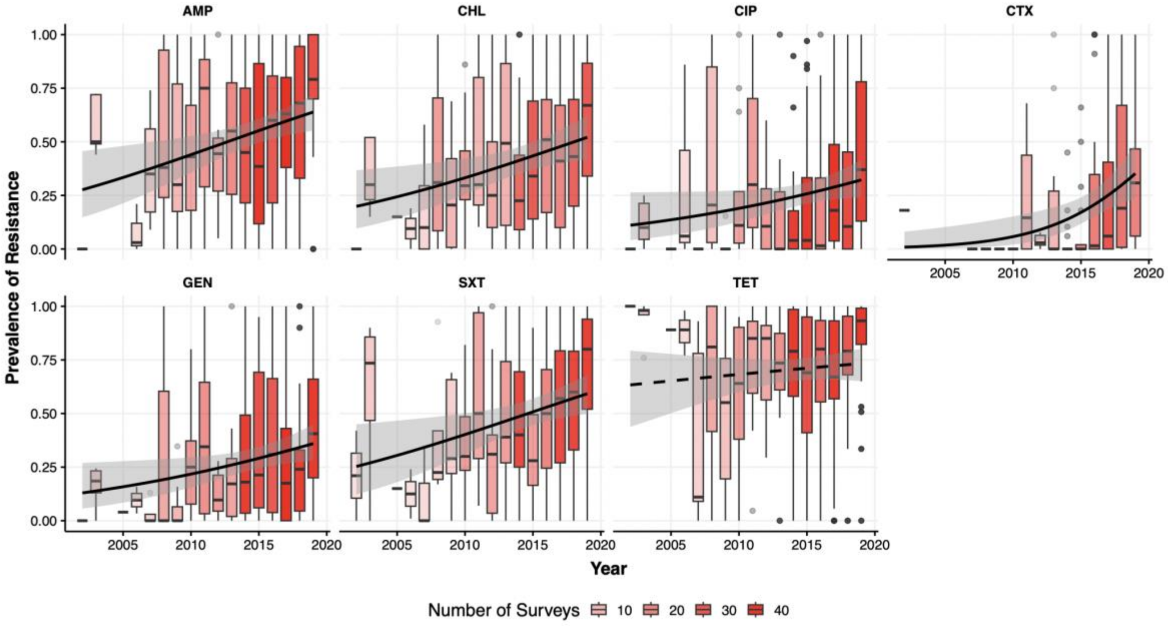

Supplementary Figure 2. **Pigs**: temporal trends of the prevalence of resistance, for ampicillin (AMP, n = 316, p = 0.0038), chloramphenicol (CHL, n = 242, p = 0.010), ciprofloxacin (CIP, n = 301, p = 0.049), cefotaxime (CTX, n = 182, p = 0.0027), gentamicin (GEN, n = 325, p = 0.024), sulfamethoxazole-trimethoprim (SXT, n = 255, p = 0.012), and tetracycline (TET, n = 307, p = 0.39). Solid lines represent significant temporal trends ($p < 0.05$), and dashed lines represent nonsignificant trends. Transparency levels of the red colors were proportional to the number of surveys published each year. Temporal trends were significant (p value < 0.05) for all antimicrobials except TET.

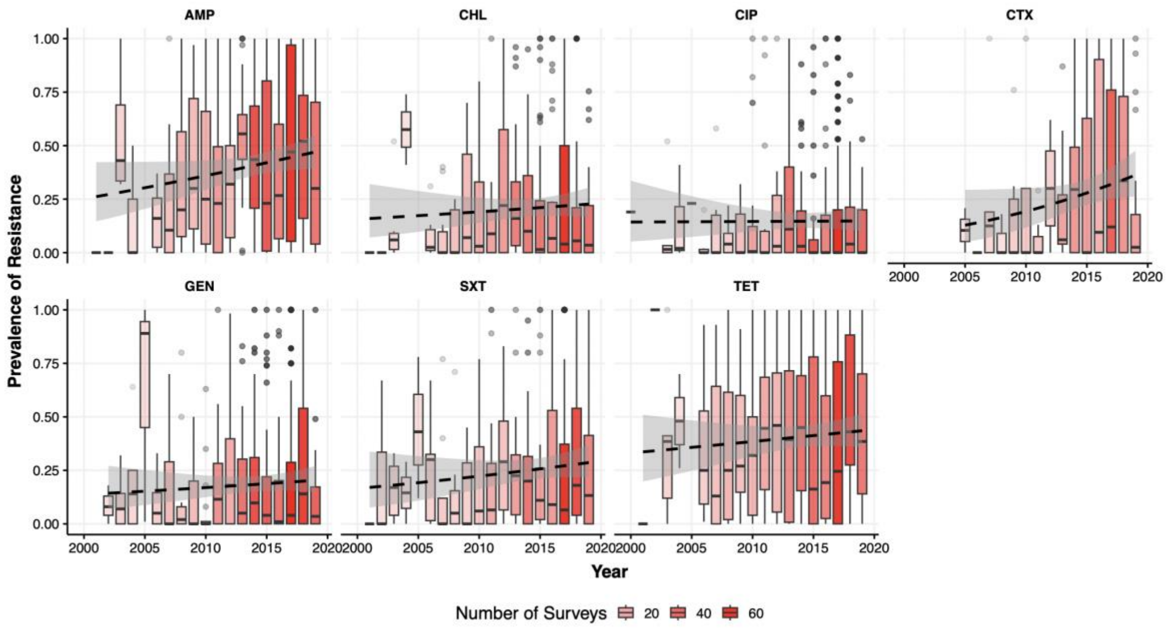

Supplementary Figure 3. **Cattle**: temporal trends of the prevalence of resistance, for ampicillin (AMP, n = 402, p = 0.057), chloramphenicol (CHL, n = 377, p = 0.47), ciprofloxacin (CIP, n = 395, p = 0.96), cefotaxime (CTX, n = 218, p = 0.05), gentamicin (GEN, n = 452, p = 0.43), sulfamethoxazole-trimethoprim (SXT, n = 323, p = 0.25), and tetracycline (TET, n = 380, p = 0.38). Solid lines represent significant temporal trends ($p < 0.05$), and dashed lines represent nonsignificant trends. Transparency levels of the red colors were proportional to the number of surveys published each year. Temporal trends were not significant ($p > 0.05$) for all antimicrobials.

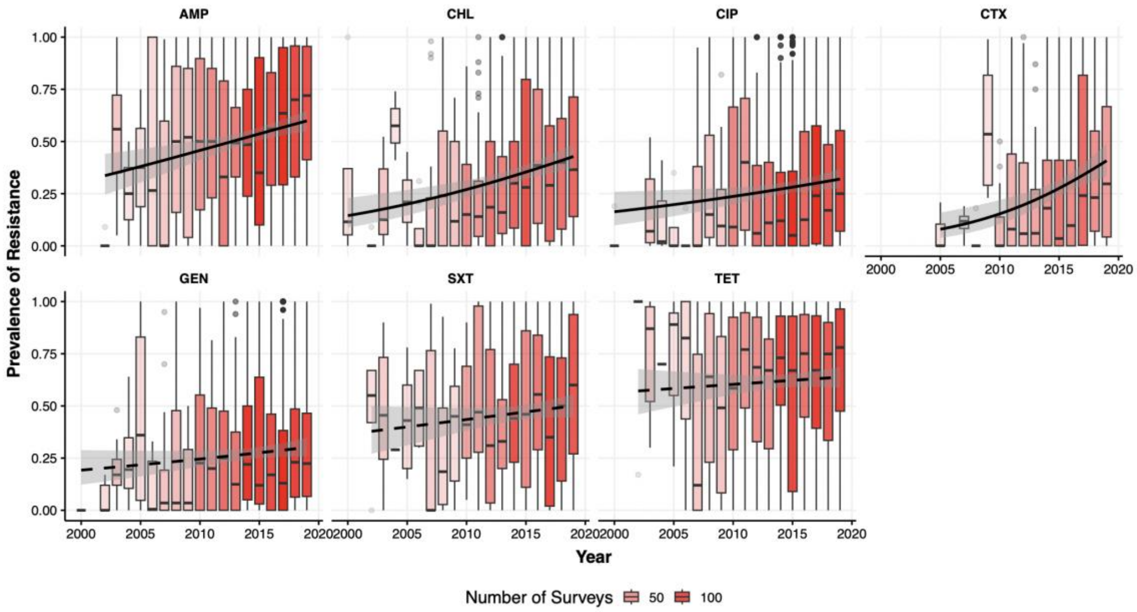

Supplementary Figure 4. **Asia**: temporal trends of the prevalence of resistance for ampicillin (AMP, n = 1,008, p = 0.00029), chloramphenicol (CHL, n = 825, p = 0.000073), ciprofloxacin (CIP, n = 1,023, p = 0.020), cefotaxime (CTX, n = 508, p = 0.000069), gentamicin (GEN, n = 1,087, p = 0.088), sulfamethoxazole-trimethoprim (SXT, n = 778, p = 0.13), and tetracycline (TET, n = 919, p = 0.36). Solid lines represent significant temporal trends ($p < 0.05$), and dashed lines represent nonsignificant trends. Transparency levels of the red colors were proportional to the number of surveys published each year. Temporal trends were significant ($p < 0.05$) for AMP, CHL, CIP, and CTX.

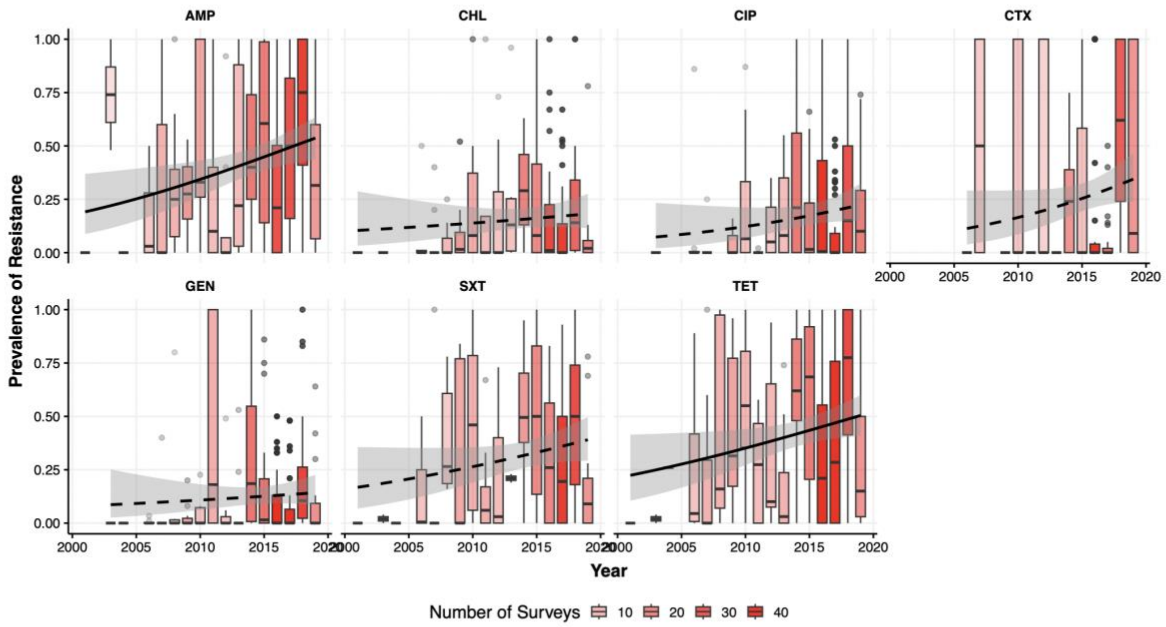

Supplementary Figure 5. **Africa**: temporal trends of the prevalence of resistance for ampicillin (AMP, n = 251, p = 0.0086), chloramphenicol (CHL, n = 244, p = 0.43), ciprofloxacin (CIP, n = 245, p = 0.13), cefotaxime (CTX, n = 179, p = 0.066), gentamicin (GEN, n = 268, p = 0.50), sulfamethoxazole-trimethoprim (SXT, n = 219, p = 0.081), and tetracycline (TET, n = 267, p = 0.034). Solid lines represent significant temporal trends ($p < 0.05$), and dashed lines represent nonsignificant trends. Transparency levels of the red colors were proportional to the number of surveys published each year. Temporal trends were significant ($p < 0.05$) for TET and AMP.

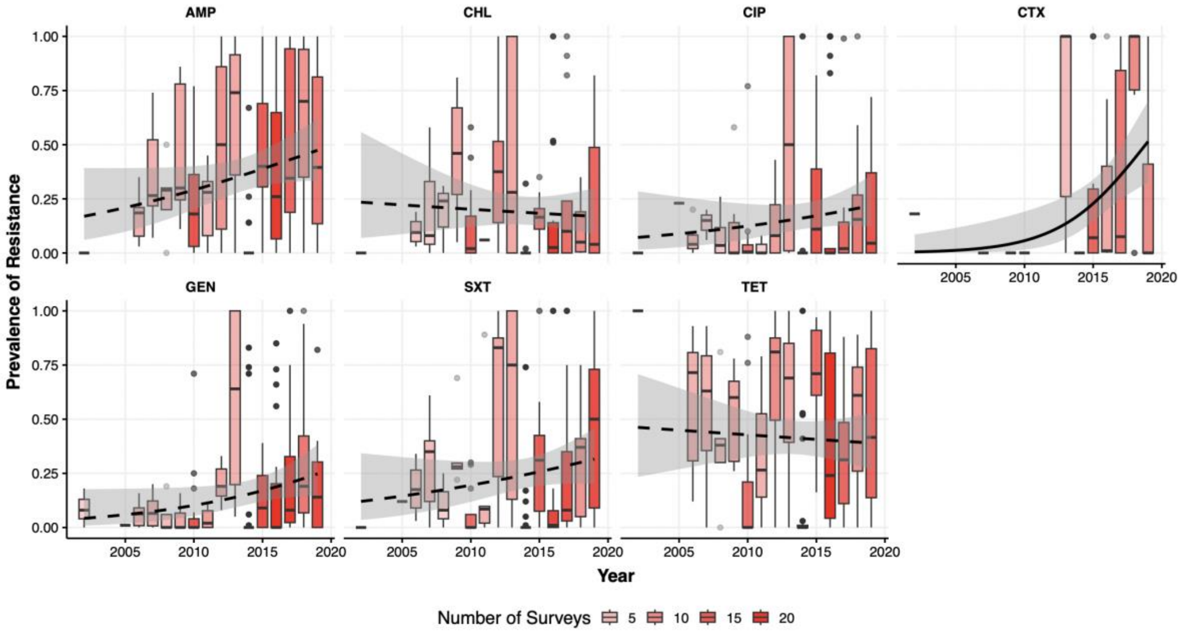

Supplementary Figure 6. **America**: temporal trends of the prevalence of resistance for ampicillin (AMP, n = 152, p = 0.065), chloramphenicol (CHL, n = 140, p = 0.68), ciprofloxacin (CIP, n = 152, p = 0.23), cefotaxime (CTX, n = 93, p = 0.0029), gentamicin (GEN, n = 177, p = 0.051), sulfamethoxazole-trimethoprim (SXT, n = 153, p = 0.18), and tetracycline (TET, n = 156, p = 0.69). Solid lines represent significant temporal trends ($p < 0.05$), and dashed lines represent nonsignificant trends. Transparency levels of the red colors were proportional to the number of surveys published each year. Temporal trends were significant ($p < 0.05$) for CTX.

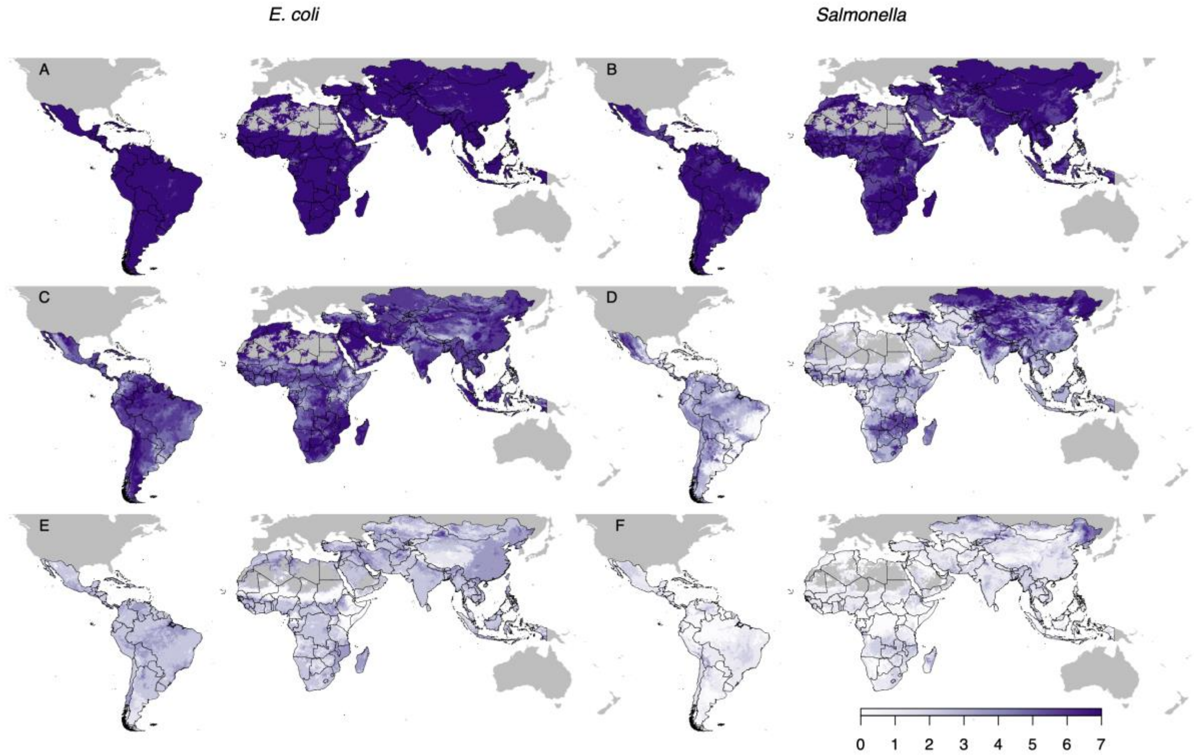

Supplementary Figure 7. The number of antimicrobials (out of 7) with resistance higher than 10% (N10: A, B), 25% (N25: C, D) and 50% (N50: E, F) in *E. coli* and *Salmonella*.

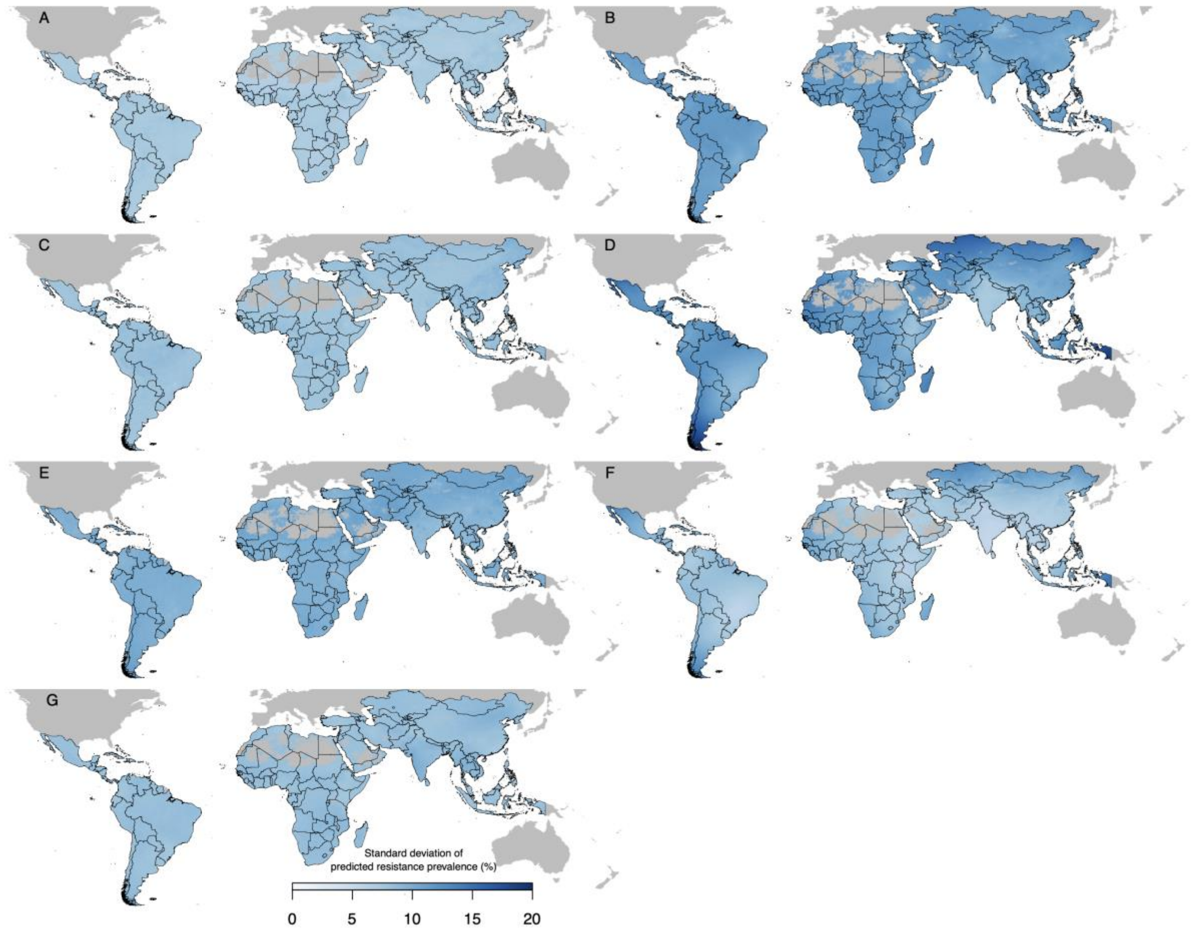

Supplementary Figure 8. Uncertainty of the predictions of resistance prevalence in *E. coli*, for tetracycline (TET), ampicillin (AMP), sulfamethoxazole-trimethoprim (SXT), chloramphenicol (CHL), ciprofloxacin (CIP), gentamicin (GEN), and cefotaxime (CTX). Shades of blue indicates the standard deviation on the predictions of resistance prevalence for each antimicrobial.

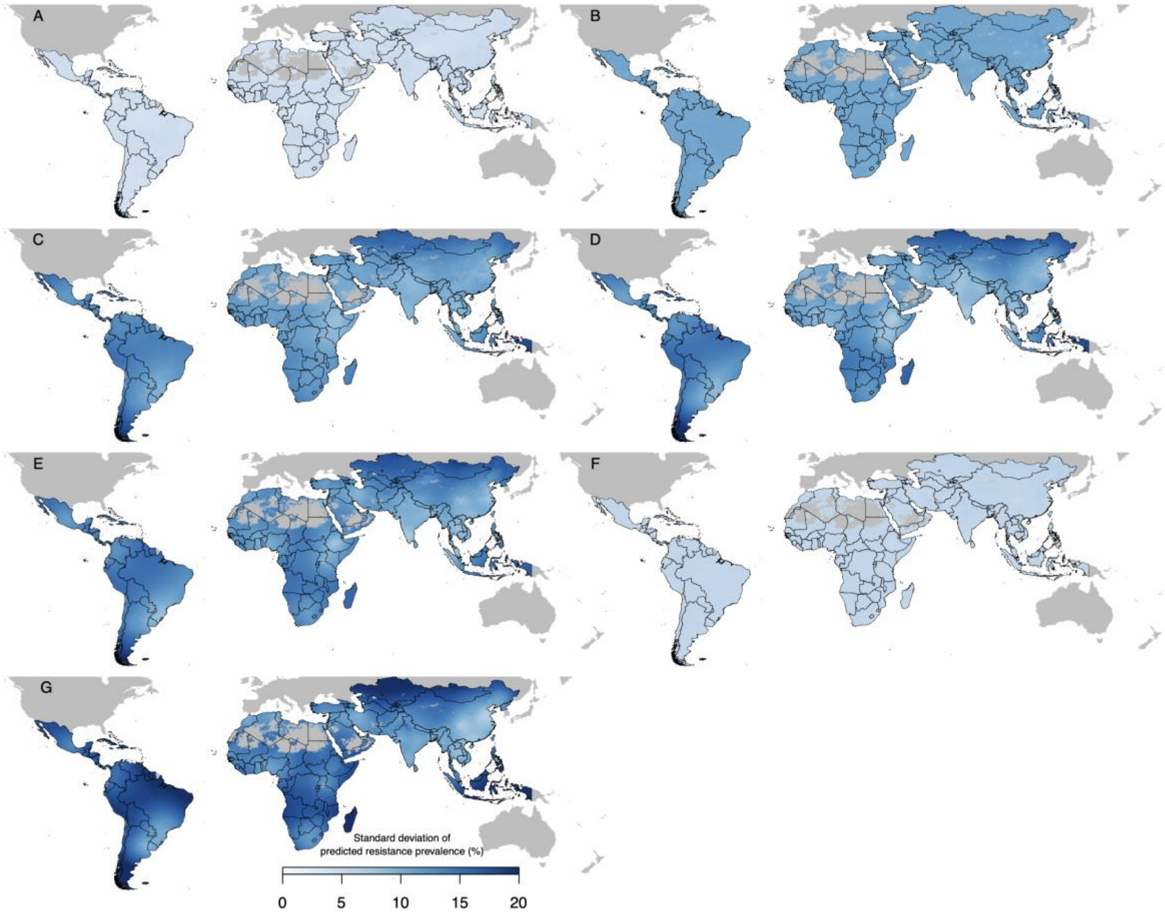

Supplementary Figure 9. Uncertainty of the predictions of resistance prevalence in *Salmonella*, for tetracycline (TET), ampicillin (AMP), sulfamethoxazole-trimethoprim (SXT), chloramphenicol (CHL), ciprofloxacin (CIP), gentamicin (GEN), and cefotaxime (CTX). Shades of blue indicates the standard deviation on the predictions of resistance prevalence for each antimicrobial.

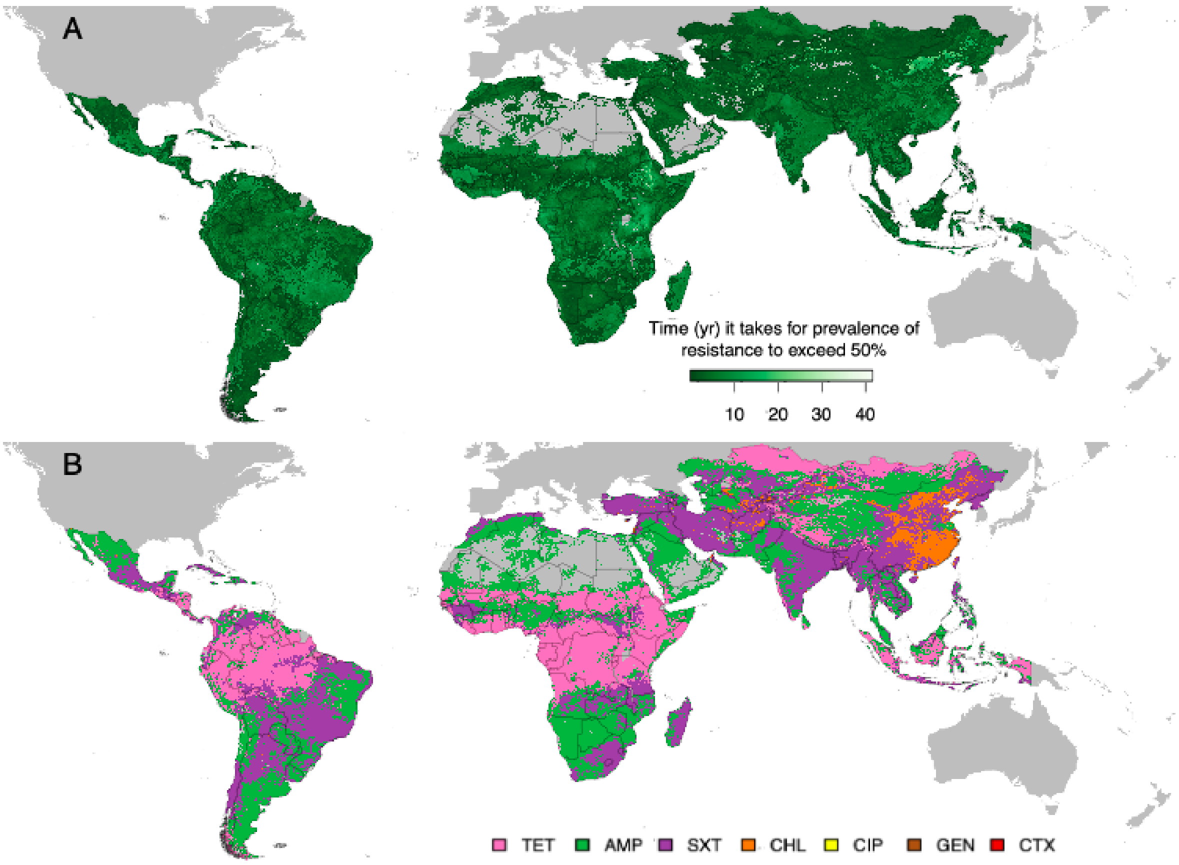

Supplementary Figure 10. Estimated time (years) that it takes for the prevalence of resistance to exceed 50% (A), for the predicted antimicrobial with the highest probability of its resistance prevalence exceeding 50% in the future (B).

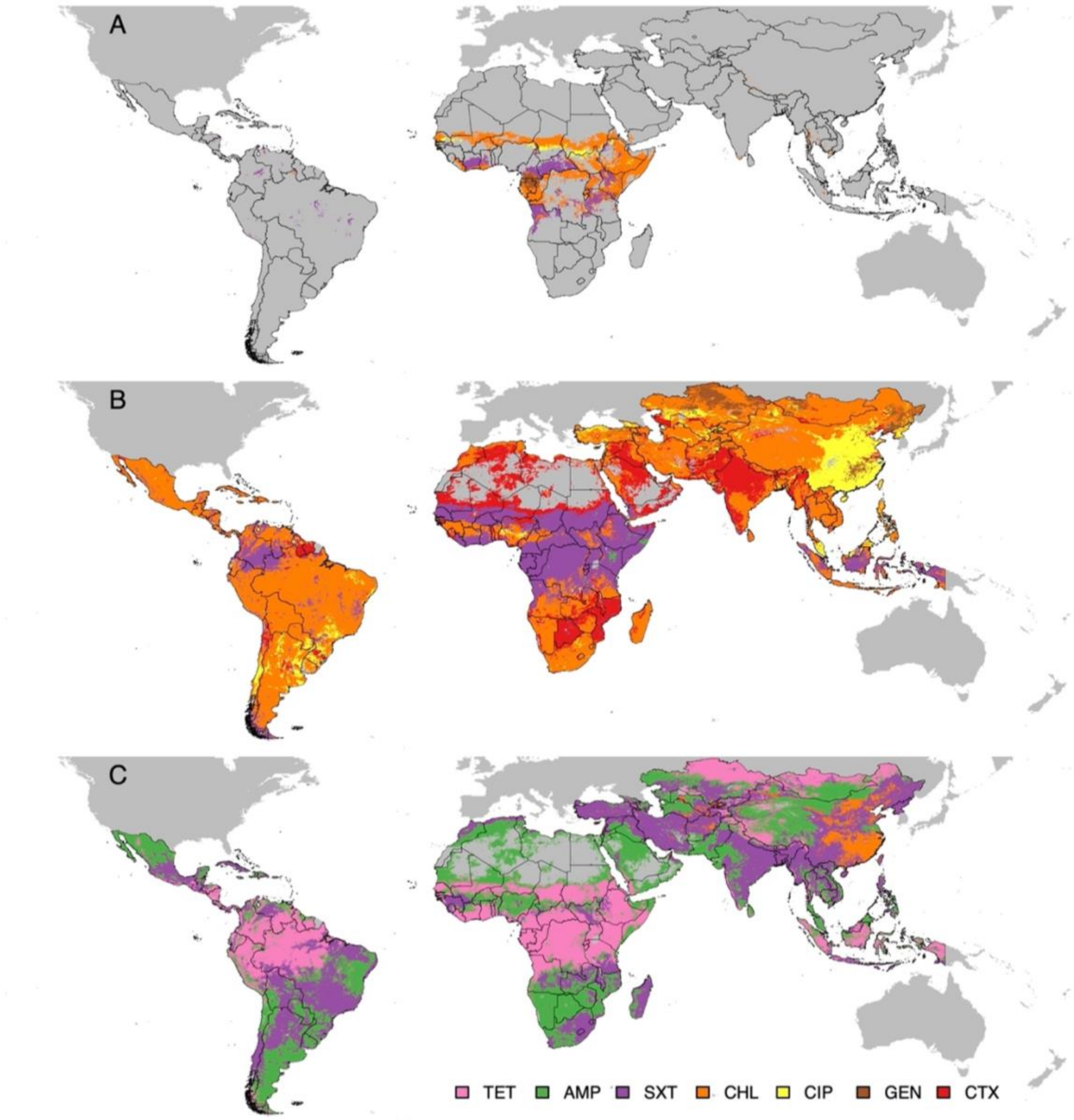

Supplementary Figure 11. Geographic distribution of antimicrobials with the highest probability of their resistance prevalence exceeding critical levels (A: 20%; B: 35%; C: 50%) in the future. TET: tetracycline; AMP: ampicillin; SXT: sulfamethoxazole-trimethoprim; CHL: chloramphenicol; CIP: ciprofloxacin; GEN: gentamicin; CTX: cefotaxime.

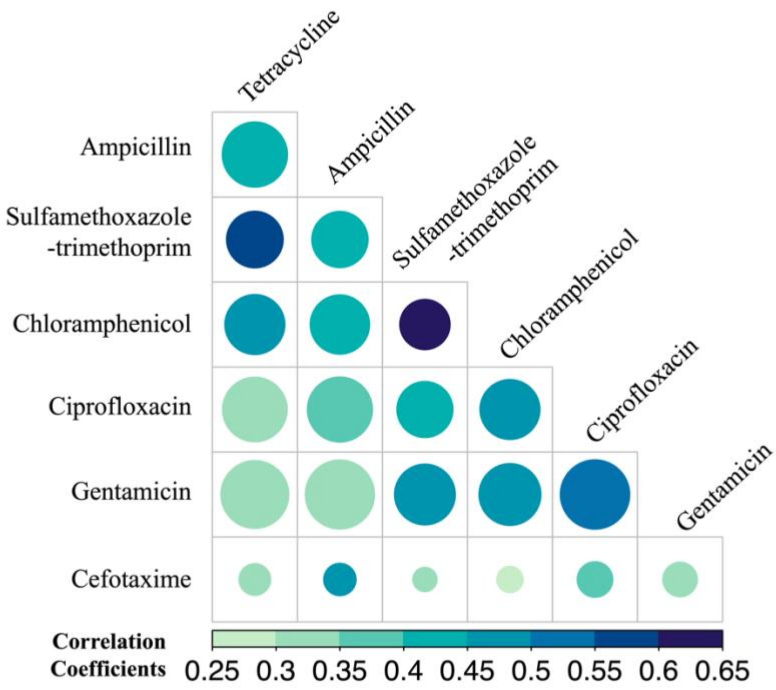

Supplementary Figure 12. Correlation coefficients between prevalence of antimicrobial resistance for 7 antimicrobial classes across 1,015 point prevalence surveys from food animals. Circle sizes are proportional to sample sizes.

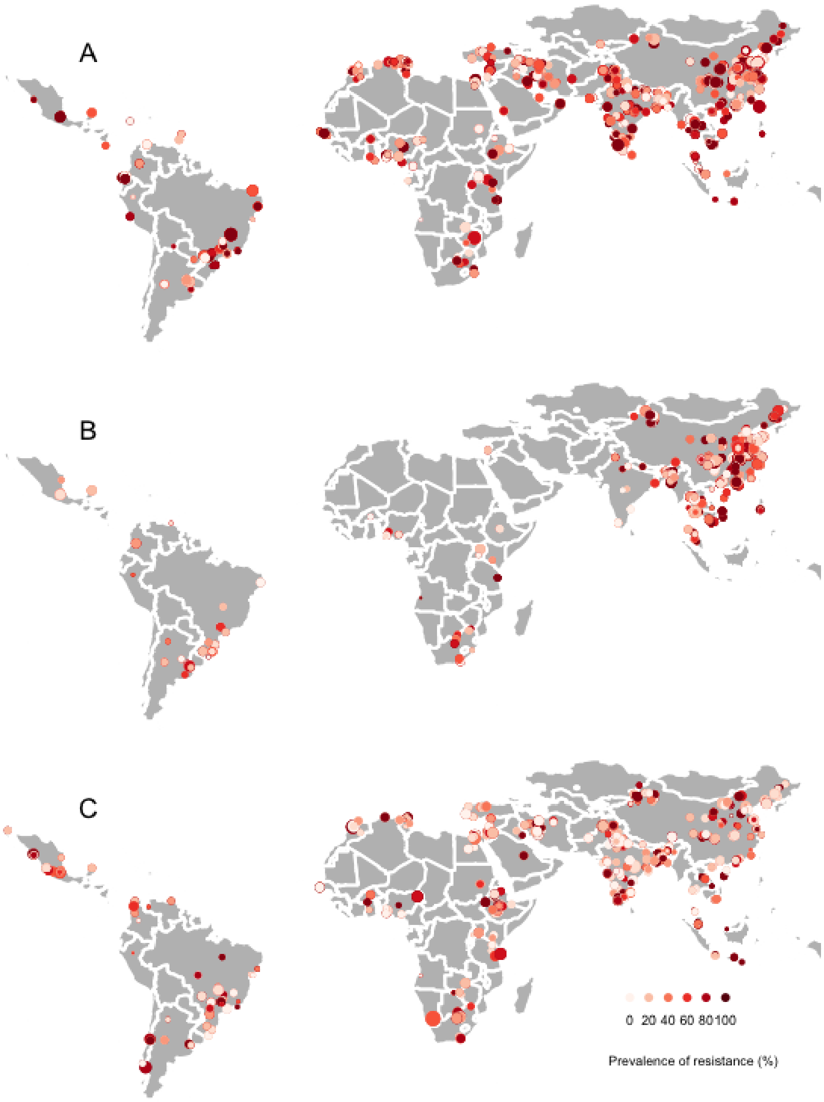

Supplementary Figure 13. Geographic locations of point-prevalence surveys reporting resistance prevalence of *E. coli* and *Salmonella* isolated from poultry (A), pigs (B), and cattle (C). Sizes of the circle were in proportion to the log10 transformed sample sizes of each survey. Colors of the circles represented the prevalence of resistance reported in each survey.

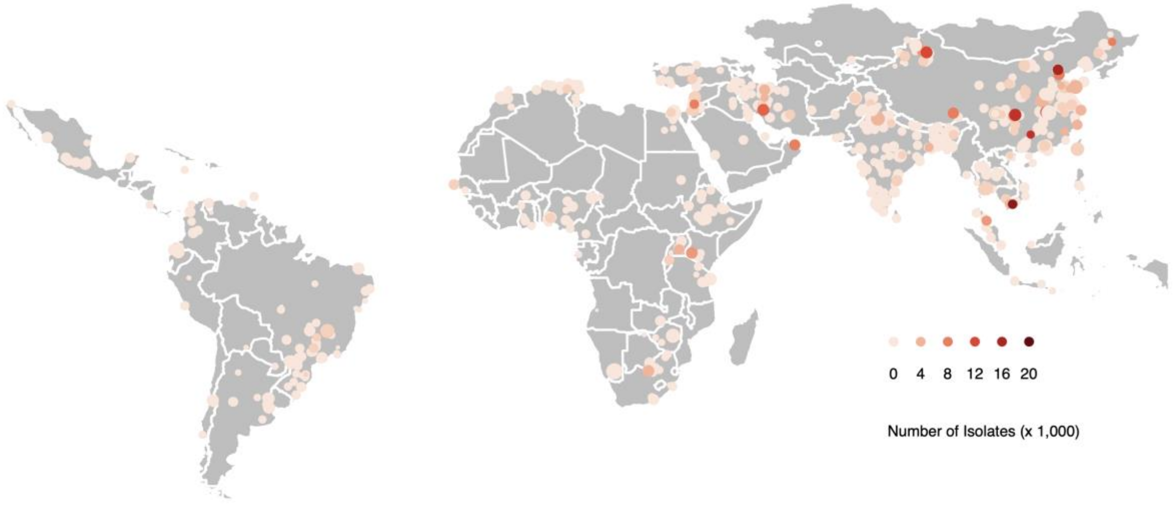

Supplementary Figure 14. Geographic locations of point-prevalence surveys reporting resistance prevalence of *E. coli* and *Salmonella*. Sizes of the circle were in proportion to the log10 transformed sample sizes of each survey. Colors of the circles represented the number of bacterial isolates used to test the prevalence of resistance in each survey. The average number of isolates in each survey was 71 in Africa, 98 in America, and 94 in Asia.

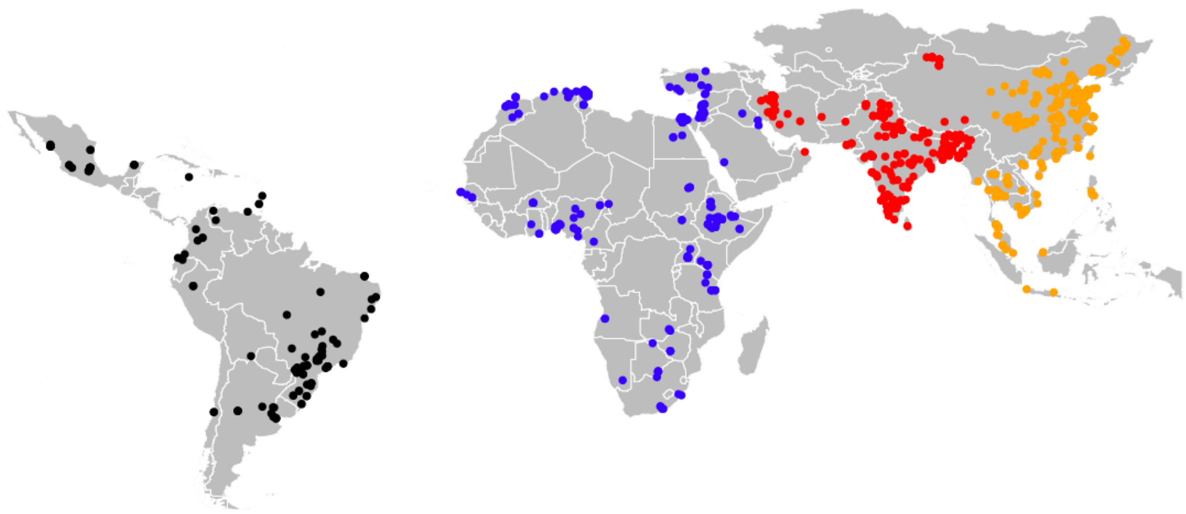

Supplementary Figure 15. Distribution of spatial folds used for the four-fold spatial cross-validation procedure of the child models.

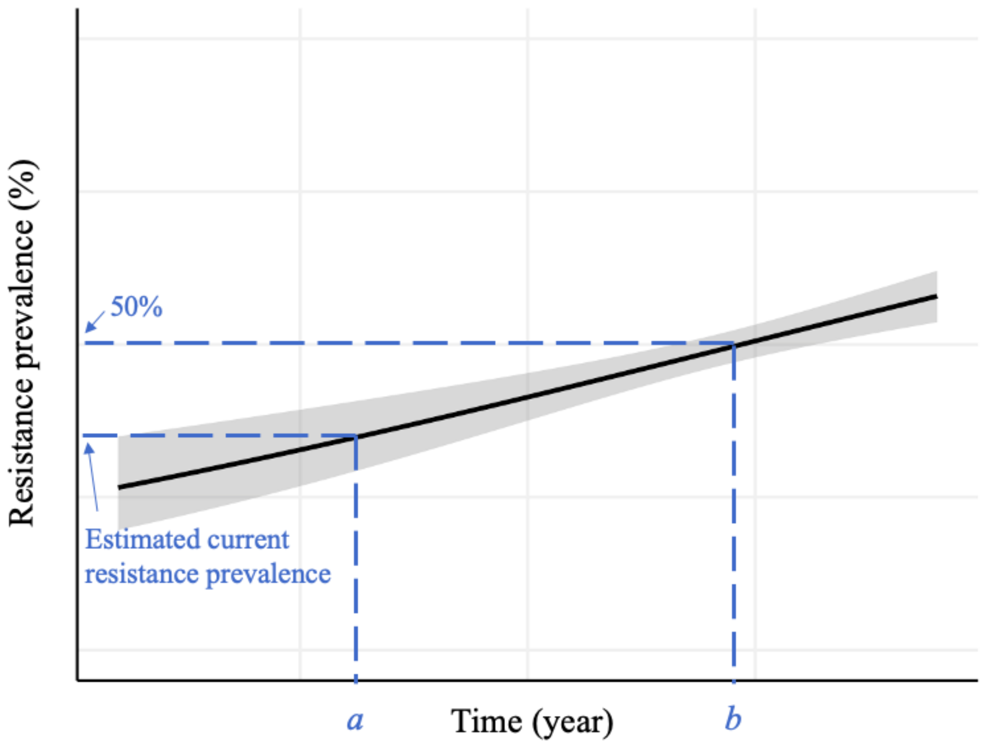

Supplementary Figure 16. Illustration of the estimation of the time it takes for resistance prevalence of an antimicrobial to reach 50%. Time point ‘*a’* is associated with the estimated current resistance prevalence at a 10x10 km pixel; time point ‘*b’* is associated with 50% resistance. The difference between ‘*a*’ and ‘*b*’ is the estimated time for resistance prevalence to exceed 50%.

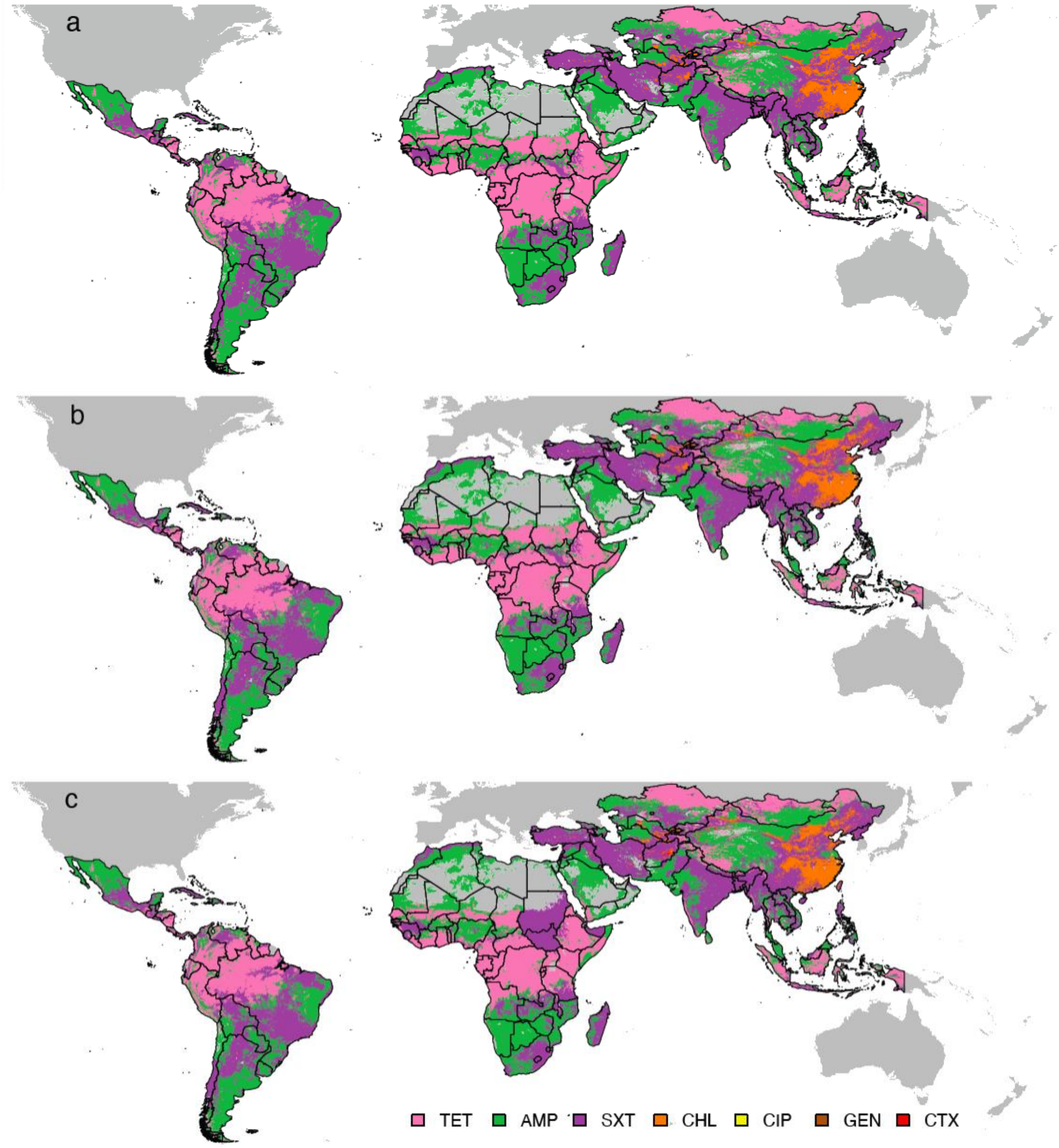

Supplementary Figure 17. Geographic distribution of antimicrobials with the highest probability of their resistance prevalence exceeding 50% in the future, with missing resistance prevalence in each survey imputed using LASSO regression (a), Bayesian linear regression (b), and feed-forward neural network (c). TET: tetracycline; AMP: ampicillin; SXT: sulfamethoxazole-trimethoprim; CHL: chloramphenicol; CIP: ciprofloxacin; GEN: gentamicin; CTX: cefotaxime.

Supplementary Tables

Supplementary Table 1. Extraction of point prevalence surveys (PPS) of antimicrobial resistance in *E. coli* and *Salmonella*, and exclusion criteria.

Exclusion Criteria	Literature Review Round #1	Literature Review Round #2	Literature Review Round #3
	n_{hits} = 32,030	n_{hits} = 8,481	n_{hits} = 3,814
Reviews, meta-analysis, and other non-PPS studies	- 30,038	- 7,401	- 3,263
	$n_{screened}$ = 1,992	$n_{screened}$ = 1,080	$n_{screened}$ = 551
Strain Surveys	NA	-115	-33
Diseased Animals	NA	-164	-21
Mixed Samples	NA	-97	-92
No Geographic Data	NA	-13	-54
Others	NA	-370	-238
	n_{PPS} = 926	n_{PPS} = 321	n_{PPS} = 113
Small Species Sample Size	-25	-6	-4
Drug-Pathogen Combinations Not Considered	-156	-45	-36
	n_{PPS_used} = 745	n_{PPS_used} = 270	n_{PPS_used} = 73

Supplementary Table 2. Coefficients associated with logistic regressions on temporal trends of resistance prevalence for Africa, Asia and America. Significant ($p < 0.05$) coefficient values are shown in bold. No adjustments are made for multiple comparisons.

	All		Africa (n = 1,673)		Asia (n = 6,148)		America (1,023)	
	estimate	standard error	estimate	standard error	estimate	standard error	estimate	standard error
TET	0.027	0.014	**0.07**	**0.033**	0.016	0.018	-0.017	0.043
AMP	**0.074**	**0.015**	**0.088**	**0.034**	**0.063**	**0.018**	0.087	0.047
SXT	**0.045**	**0.016**	0.064	0.037	0.029	0.019	0.071	0.053
CHL	**0.058**	**0.017**	0.036	0.046	**0.078**	**0.02**	-0.024	0.059
CIP	**0.051**	**0.018**	0.081	0.054	**0.046**	**0.02**	0.076	0.063
GEN	**0.037**	**0.016**	0.035	0.053	0.031	0.018	0.118	0.061
CTX	**0.155**	**0.03**	0.109	0.059	**0.148**	**0.037**	**0.319**	**0.107**

Supplementary Table 3. Environmental and anthropogenic covariates.

Name	Acronym	Year	Original Resolution	Source	Unit
Travel time to cities	acc	2015	30-arcsec resolution	Weiss et al 2018 [10] https://www.map.ox.ac.uk/accessibility_to_cities/.	minute
Antimicrobial use in animals 2013	use_2013	2013	0.083333 decimal degrees	Van Boeckel et al 2017 [11] http://science.sciencemag.org/content/357/6358/1350.full	Log10[(mg/pixel)+1]
Antimicrobial use in animals 2020	use_2020	2020	0.083333 decimal degrees	Mulchandani et al 2023[4] https://journals.plos.org/globalpublichealth/article?id=10.1371/journal.pgph.0001305	Log10[(mg/pixel)+1]
Yearly average of minimum monthly temperature	tmp	1970-2000	2.5 minutes	Worldclim [12] http://worldclim.org/version2	°C * 10
Percentage Irrigated areas	irg	2005	0.083333 decimal degrees	Global Map of Irrigation Areas (GMIA) [13] http://www.fao.org/aquastat/en/geospatial-information/ global-maps-irrigated-areas/latest-version/	%
Population density of cattle, chickens, pigs, and sheep	ca_v4 ch_v4 pg_v4 sh_v4	2015	0.083333 decimal degrees	Gridded Livestock of the World v4 https://www.nature.com/articles/sdata2018227	Log10[(Heads/pixel)+1]
Percentage of tree coverage	veg	2013	0.083333 decimal degrees	Hansen et al 2013 [14] https://earthenginepartners.appspot.com/science-2013-global-forest/download_v1.2.html	%
Average pesticide application rate	pest	2015	0.083333 decimal degrees	PEST-CHEMGRIDS [15] https://sedac.ciesin.columbia.edu/data/set/ferman-v1-pest-chemgrids	kg/ha per year
Atmospheric ammonia	amm	2008-2016	0.01 decimal degrees	Van Damme et al 2018 [16] https://www.nature.com/articles/s41586-018-0747-1	10^{16} molecules cm^{-2}
Gross Domestic Product (GDP) in Purchasing Power Parity	gdp	2005	1 decimal degrees	G-Econ [17] https://sedac.ciesin.columbia.edu/data/set/spatialecon-gecon-v4	Billion US dollars
Fourier coefficients of Precipitation	wd1920	2001-2019	0.0083333 decimal degrees	Scharlemann et al 2008 [18] https://journals.plos.org/plosone/article?id=10.1371/journal.pone.0001408	NA
Fourier coefficients of Middle Infra-red	wd1903	2001-2019	0.0083333 decimal degrees	Scharlemann et al 2008 [18] https://journals.plos.org/plosone/article?id=10.1371/journal.pone.0001408	NA
Fourier coefficients of Normalised Difference Vegetation Index	wd1914	2001-2019	0.0083333 decimal degrees	Scharlemann et al 2008 [18] https://journals.plos.org/plosone/article?id=10.1371/journal.pone.0001408	NA
Fourier coefficients of Enhanced Vegetation	wd1915	2001-2019	0.0083333 decimal degrees	Scharlemann et al 2008 [18] https://journals.plos.org/plosone/article?id=10.1371/journal.pone.0001408	NA

Index					
Fourier coefficients of Day Land Surface Temperature	wd1907	2001-2019	0.0083333 decimal degrees	Scharlemann et al 2008 [18] https://journals.plos.org/plosone/article?id=10.1371/journal.pone.0001408	NA
Fourier coefficients of Night Land Surface Temperature	wd1908	2001-2019	0.0083333 decimal degrees	Scharlemann et al 2008 [18] https://journals.plos.org/plosone/article?id=10.1371/journal.pone.0001408	NA

Supplementary Table 4. The average estimated time for resistance prevalence to exceed 50% across all pixels on the map, for each antimicrobial class and weighted by the distribution of animals' biomass.

Antimicrobial Class	Time for resistance prevalence to exceed 50% (years)
Tetracyclines	5.1
Penicillins	1.7
Sulfonamides	7.1
Amphenicols	10.8
Quinolones	12.4
Aminoglycosides	NA
Cephalosporins	4.1

Supplementary Table 5. Coefficients of LASSO regressions predicting the possibility that resistance prevalence of an antimicrobial will exceed 50% in the future, given the preceding resistance profile. The antimicrobials included cefotaxime (CTX), sulfamethoxazole-trimethoprim (SXT), chloramphenicol (CHL), tetracycline (TET), ampicillin (AMP), ciprofloxacin (CIP), and gentamicin (GEN). Proportion_PPS: the proportion of point prevalence surveys reporting an increased resistance prevalence to over 50% for an antimicrobial, out of all alternative antimicrobials; Proportion_AMU: the proportion of usage (kg) of an antimicrobial out of all alternative antimicrobials; N50: the number of antimicrobials with resistance above 50% in the preceding resistance profile. The abbreviations of the other covariates are explained in Supplementary Table 3. Covariates for which coefficients were 0 for all antimicrobials were removed from the table.

	TET	AMP	SXT	CHL	CIP	GEN	CTX
(Intercept)	-7.63	-2.921	-3.624	-5.311	-4.069	-4.183	-3.628
proportion_PPS	19.36	5.497	2.151	1.734	-0.96	-0.915	-1.215
proportion_AMU	0	0	0.346	0	0	0	0
N50	0.013	0.364	0.936	1.103	1	0.934	0.989
use_2020	0	0	0	0.079	0	0	-0.045
use_2013	0	0	0.01	0.004	-0.007	0	-0.215
ch_v4	0	0	0	0	0	0	-0.076
sh_v4	0	0	-0.022	0	0	0.039	0
Pest	0	0	0.002	0	0	-0.013	-0.001
amm	0	0	0	-0.004	0	0	0.056
gdp	0	0	0	0.058	0	0	-0.134
wd1920a0	0	0	0	0	-0.121	0	0
wd1920a3	-0.096	0	0	0	0	0	0
wd1920d2	0	0	0	-0.014	0.117	0	0
wd1920d3	0	0.11	-0.079	-0.057	0	0	0
wd1920dd	0	0	0	0	0.035	0	0
wd1920mn	0	-0.066	0	0.003	0	0	0
wd1920vr	0	0	0	0.016	0	0	0
wg1903a1	0	0	0	-0.095	0	0	0
wg1903a2	0	0	0	0	0.027	0	0
wg1903d3	0	0	0	0	0.084	0	0
wg1903dd	0	0	0	0	-0.176	0	0
wg1903mn	0	0.17	-0.073	0	0	0	0
wg1903vr	0	0	0	0	0	0	0.054
wg1907a0	0	0.134	0	0	0	0	0
wg1907a2	0	0	0	-0.026	0	0.002	0
wg1907a3	0	0	0	0	0	0.091	0
wg1907d2	0	0	-0.072	0	0	0	0
wg1907d3	0	-0.002	0	0.008	0	0	0
wg1907mn	0	0	-0.083	0	0	0	0
wg1908a2	0	0	0	0	0	0.176	0
wg1908a3	0	0	0.08	0.099	0	0	-0.307
wg1908d2	0	0	0	-0.058	0	0	0
wg1914da	0	0	0	0	0	0.102	0.004
wg1914dd	0	0	0	-1.562	0	0	2.818
wg1914mx	0	0	0	-0.163	0	0	0
wg1914vr	0	0	0	0	0.119	0	0

wg1915a2	0.03	0	0	-0.059	0	0	0
wg1915d1	-0.051	0	0	0	0	0	0
wg1915d3	0.086	0	0	0	0	0	0

Supplementary Table 6. The top 20 antimicrobial compounds with reported resistance prevalence and the corresponding antimicrobial classes in point prevalence surveys.

Antimicrobial Compound	Number of Reported Resistance Prevalence	Antimicrobial Class
Gentamicin	1,452	Aminoglycosides
Ampicillin	1,347	Penicillins
Ciprofloxacin	1,345	Quinolones
Tetracycline	1,271	Tetracyclines
Chloramphenicol	1,133	Amphenicols
Sulfamethoxazole-Trimethoprim	1,068	Sulfonamides
Streptomycin	937	Aminoglycosides
Nalidixic acid	852	Quinolones
Cefotaxime	726	Cephalosporins
Amikacin	688	Aminoglycosides
Kanamycin	664	Aminoglycosides
Amoxicillin-Clavulanic acid	614	Penicillins
Ceftriaxone	521	Cephalosporins
Enrofloxacin	473	Quinolones
Ceftazidime	456	Cephalosporins
Amoxicillin	454	Penicillins
Norfloxacin	449	Quinolones
Cefalotin	351	Cephalosporins
Colistin	330	Polymixins

Supplementary Table 7. Estimated parameters of the fitted INLA models predicting the geographic distribution of resistance prevalence of each antimicrobial. The table showed the mean value and standard deviation for the range of the spatial random effect, and the coefficients of three child models. The antimicrobials included cefotaxime (CTX), sulfamethoxazole-trimethoprim (SXT), chloramphenicol (CHL), tetracycline (TET), ampicillin (AMP), ciprofloxacin (CIP), and gentamicin (GEN).

	CTX	SXT	CHL	TET	AMP	CIP	GEN
range.mean	3.39	5.65	3.82	3.25	2.4	4.52	3.35
range.sd	1.58	1.31	1.47	1.57	1.52	3.92	1.5
beta_BRT.mean	0.47	0.36	0.38	0.25	0.29	0.34	0.45
beta_BRT.sd	0.06	0.06	0.05	0.05	0.04	0.04	0.06
beta_LASSO.mean	0.4	0.41	0.37	0.39	0.42	0.32	0.36
beta_LASSO.sd	0.06	0.05	0.05	0.04	0.05	0.04	0.05
beta_NNR.mean	0.41	0.31	0.36	0.37	0.35	0.43	0.39
beta_NNR.sd	0.06	0.04	0.05	0.05	0.05	0.05	0.05

Supplementary Table 8. Common resistance profiles in point prevalence surveys, with 2, 3, and 4 antimicrobials with resistance higher than 50% (N50). The total number of surveys for each N50 category, and the number of surveys reporting each resistance profile were shown in the brackets.

Number of antimicrobials with resistance higher than 50%	Resistance profiles
N50 = 2 (n = 201)	TET-AMP (n=97)<br>TET-SXT (n=45)
N50 = 3 (n = 161)	TET-AMP-SXT (n=60)<br>TET-SXT-CHL (n=25)<br>TET-AMP-CIP (n=17)<br>TET-AMP-CTX (n=14)
N50 = 4 (n = 138)	TET-AMP-SXT-CHL (n=66)<br>TET-AMP-SXT-CIP (n=12)<br>TET-SXT-CHL-GEN (n=11)<br>TET-AMP-SXT-CTX (n=8)

Supplementary Table 9. Number of point-prevalence surveys conducted on chicken, pigs, and cattle in each year.

Year	Chicken	Pigs	Cattle
**2000**	1	0	1
**2001**	0	0	1
**2002**	2	2	2
**2003**	10	4	6
**2004**	0	0	5
**2005**	2	1	2
**2006**	6	3	6
**2007**	12	4	11
**2008**	11	12	14
**2009**	12	8	11
**2010**	31	14	18
**2011**	17	16	22
**2012**	50	12	19
**2013**	33	16	34
**2014**	63	38	46
**2015**	56	32	42
**2016**	85	31	33
**2017**	70	39	60
**2018**	50	31	41
**2019**	59	40	35

Supplementary Table 10. Number of point-prevalence surveys conducted on chicken, pigs, and cattle in each country.

Country ISO3	Chicken	Pigs	Cattle
AGO	1	1	1
BFA	2	1	2
BWA	1	1	3
CMR	1	0	0
DZA	8	0	2
EGY	17	0	12
ETH	11	2	26
GAB	1	0	0
GHA	3	0	2
GMB	1	0	0
KEN	5	2	2
MAR	6	0	4
NAM	0	0	1
NGA	16	4	10
SDN	0	0	2
SEN	1	0	1
TCD	1	0	0
TUN	13	0	8
TZA	3	1	7
UGA	4	2	3
ZAF	5	6	10
ZMB	1	0	2
ZWE	2	0	1
RWA	1	0	0
IND	84	20	93
BGD	36	0	11
NPL	8	1	1
BTN	1	1	0
PAK	10	0	4
IRN	33	0	21
IRQ	6	0	2
ISR	2	0	2
LBN	3	1	4
QAT	1	0	0
OMN	1	0	0
JOR	0	0	3
SAU	2	0	1
TUR	11	0	8
ARG	5	8	5
BOL	1	0	0
BRA	28	13	27
COL	1	1	4
ECU	6	0	0
PER	2	1	1
VEN	1	1	2

**CHL**	0	0	3
**CRI**	1	0	0
**MEX**	6	5	15
**GRD**	2	0	0
**LCA**	0	0	0
**JAM**	1	0	0
**CHN**	169	186	75
**IDN**	3	0	5
**KHM**	2	3	0
**LAO**	0	2	2
**MYS**	6	2	4
**MMR**	1	0	0
**PHL**	1	3	0
**SGP**	1	0	0
**THA**	14	26	10
**VNM**	14	9	7
**LKA**	1	0	0
**KWT**	1	0	0
**PRY**	1	0	0

Supplementary Table 11. The percentage of 10 x 10 km pixels in each country that have an uncertainty of the predicted priority antimicrobial above 40%.

Country ISO3	Percentage of pixels with uncertainty > 40%	Country ISO3	Percentage of pixels with uncertainty > 40%	Country ISO3	Percentage of pixels with uncertainty > 40%
AFG	21%	GNB	16%	PAK	8%
AGO	3%	GNQ	0%	PAN	11%
ARE	15%	GTM	10%	PER	14%
ARG	3%	GUY	12%	PHL	16%
ARM	15%	HND	3%	PRI	6%
AZE	6%	HTI	9%	PRK	77%
BDI	7%	IDN	14%	PRY	3%
BEN	8%	IND	7%	PSX	38%
BFA	6%	IRN	13%	QAT	16%
BGD	2%	IRQ	8%	RWA	6%
BLZ	7%	ISR	34%	SAH	100%
BOL	7%	JAM	9%	SAU	4%
BRA	9%	JOR	3%	SDN	21%
BRN	16%	KAB	6%	SDS	35%
BTN	4%	KAS	100%	SEN	3%
BWA	0%	KAZ	6%	SGP	100%
CAF	6%	KEN	2%	SLE	29%
CHL	12%	KGZ	16%	SLV	6%
CHN	12%	KHM	9%	SLO	15%
CIV	5%	KWT	8%	SOM	6%
CMR	5%	LAO	6%	SUR	11%
CNM	33%	LBN	9%	SWZ	41%
COD	8%	LBR	2%	SYR	3%
COG	15%	LBY	2%	TCD	2%
COL	17%	LKA	9%	TGO	16%
CRI	12%	LSO	78%	THA	9%
CUB	13%	MAR	7%	TJK	11%
CYN	57%	MDG	10%	TKM	3%
CYP	35%	MEX	7%	TLS	11%
DJI	44%	MLI	7%	TTO	21%
DOM	6%	MMR	10%	TUN	3%
DZA	3%	MNG	9%	TUR	8%
ECU	13%	MOZ	6%	TWN	13%
EGY	3%	MRT	3%	TZA	9%
ERI	1%	MWI	10%	UGA	9%
ESB	0%	MYS	11%	URY	9%
ETH	3%	NAM	2%	UZB	10%
GAB	3%	NER	3%	VEN	9%
GEO	14%	NGA	10%	VNM	12%
GHA	5%	NIC	6%	YEM	2%
GIN	14%	NPL	6%	ZAF	4%
GMB	21%	OMN	12%	ZMB	9%
				ZWE	2%

References

1. Van Boeckel, T. P. *et al.* Global trends in antimicrobial resistance in animals in low- and middle-income countries. *Science* **365**, 1266-+ (2019).
2. Van Buuren, S. & Groothuis-Oudshoorn, K. mice: Multivariate imputation by chained equations in R. *J. Stat. Softw.* **45**, 1–67 (2011).
3. Bhatt, S. *et al.* Improved prediction accuracy for disease risk mapping using Gaussian process stacked generalization. *J. R. Soc. Interface* **14**, 20170520 (2017).
4. Mulchandani, R., Wang, Y., Gilbert, M. & Van Boeckel, T. P. Global trends in antimicrobial use in food-producing animals: 2020 to 2030. *PLOS Glob. Public Health* **3**, e0001305 (2023).
5. Elith, J., Leathwick, J. R. & Hastie, T. A working guide to boosted regression trees. *J. Anim. Ecol.* **77**, 802–813 (2008).
6. Tibshirani, R. Regression shrinkage and selection via the lasso: a retrospective. *J. R. Stat. Soc. Ser. B-Stat. Methodol.* **73**, 273–282 (2011).
7. Chollet, F. Keras. (2015).
8. Rue, H., Martino, S. & Chopin, N. Approximate Bayesian inference for latent Gaussian models by using integrated nested Laplace approximations. *J. R. Stat. Soc. Ser. B Stat. Methodol.* **71**, 319–392 (2009).
9. Zhao, C. *et al.* Geographically targeted surveillance of livestock could help prioritize intervention against antimicrobial resistance in China. *Nat. Food* **2**, 596–602 (2021).
10. Weiss, D. J. *et al.* A global map of travel time to cities to assess inequalities in accessibility in 2015. *Nature* **553**, 333–336 (2018).
11. Van Boeckel, T. P. *et al.* Global trends in antimicrobial use in food animals. *Proc. Natl. Acad. Sci.* **112**, 5649–5654 (2015).
12. Fick, S. E. & Hijmans, R. J. WorldClim 2: new 1-km spatial resolution climate surfaces for global land areas. *Int. J. Climatol.* **37**, 4302–4315 (2017).
13. Siebert, S. *et al.* Development and validation of the global map of irrigation areas. (2005).
14. Hansen, M. C. *et al.* High-resolution global maps of 21st-century forest cover change. *science* **342**, 850–853 (2013).
15. Maggi, F., Tang, F. H. M., la Cecilia, D. & McBratney, A. PEST-CHEMGRIDS, Global Gridded Maps of the Top 20 Crop-specific Pesticide Application Rates from 2015 to 2025. *Sci. Data* **6**, 1–20 (2019).
16. Van Damme, M. *et al.* Industrial and agricultural ammonia point sources exposed. *Nature* **564**, 99–103 (2018).
17. Nordhaus, W. D. Geography and Macroeconomics: New Data and New Findings. *Proc. Natl. Acad. Sci. U. S. Am. PNAS* **103**, (2005).
18. Scharlemann, J. P. *et al.* Global data for ecology and epidemiology: a novel algorithm for temporal Fourier processing MODIS data. *PloS One* **3**, e1408 (2008).
