# Supplementary material for: Global surveillance of antimicrobial resistance in food animals using priority drugs maps
Source: Nat Commun. 2024 Jan 26;15:763. doi: 10.1038/s41467-024-45111-7 (PMC10817973; doi:10.1038/s41467-024-45111-7)
Supplement: Supplementary file 3 — Reporting Summary [file 41467_2024_45111_MOESM3_ESM.pdf]

nature portfolio

Corresponding author(s): Thomas Van Boeckel

Last updated by author(s): Oct 2, 2023

Reporting Summary

Nature Portfolio wishes to improve the reproducibility of the work that we publish. This form provides structure for consistency and transparency in reporting. For further information on Nature Portfolio policies, see our Editorial Policies and the Editorial Policy Checklist.

Statistics

For all statistical analyses, confirm that the following items are present in the figure legend, table legend, main text, or Methods section.

n/a	Confirmed	
☐	☒	The exact sample size (*n*) for each experimental group/condition, given as a discrete number and unit of measurement
☒	☐	A statement on whether measurements were taken from distinct samples or whether the same sample was measured repeatedly
☐	☒	The statistical test(s) used AND whether they are one- or two-sided<br>*Only common tests should be described solely by name; describe more complex techniques in the Methods section.*
☐	☒	A description of all covariates tested
☐	☒	A description of any assumptions or corrections, such as tests of normality and adjustment for multiple comparisons
☐	☒	A full description of the statistical parameters including central tendency (e.g. means) or other basic estimates (e.g. regression coefficient) AND variation (e.g. standard deviation) or associated estimates of uncertainty (e.g. confidence intervals)
☐	☒	For null hypothesis testing, the test statistic (e.g. *F*, *t*, *r*) with confidence intervals, effect sizes, degrees of freedom and *P* value noted<br>*Give P values as exact values whenever suitable.*
☐	☒	For Bayesian analysis, information on the choice of priors and Markov chain Monte Carlo settings
☒	☐	For hierarchical and complex designs, identification of the appropriate level for tests and full reporting of outcomes
☒	☐	Estimates of effect sizes (e.g. Cohen's *d*, Pearson's *r*), indicating how they were calculated

Our web collection on statistics for biologists contains articles on many of the points above.

Software and code

Policy information about availability of computer code

Data collection	Zotero (version 5.0.96.2) and Microsoft Excel (version 16.53) were used for the literature review.
Data analysis	All data analysis was conducted using R version 4.1.1. The R codes used to generate the results are available at Zenodo (zenodo.org/record/8400343). R packages used for the data analyses include the following:<br>grDevices (version 4.1.1)<br>RColorBrewer (version 1.1-2)<br>raster (version 3.5-2)<br>dplyr (version 1.1.2)<br>tidyr (version 1.3.0)<br>INLA (version 21.02.23)<br>gridExtra (version 2.3)<br>lattice (version 0.20-45)<br>glmnet (version 4.1-3)<br>dismo (version 1.3-5)<br>keras (version 2.7.0)<br>caret (version 6.0-90)<br>data.table (version 1.14.2)<br>car (version 3.0-11)<br>mice (version 3.13.0)<br>ModelMetrics (version 1.2.2.2)<br>spatialEco (version 1.3-7)

scales (version 1.2.1)
ggplot2 (version 3.4.2)
readxl (version 1.4.3)

For manuscripts utilizing custom algorithms or software that are central to the research but not yet described in published literature, software must be made available to editors and reviewers. We strongly encourage code deposition in a community repository (e.g. GitHub). See the Nature Portfolio guidelines for submitting code & software for further information.

Data

Policy information about availability of data

All manuscripts must include a data availability statement. This statement should provide the following information, where applicable:

- Accession codes, unique identifiers, or web links for publicly available datasets
- A description of any restrictions on data availability
- For clinical datasets or third party data, please ensure that the statement adheres to our policy

All data were extracted from literature reviews of point-prevalence surveys from PubMed (https://pubmed.ncbi.nlm.nih.gov), Scopus (https://www.scopus.com), ISI Web of Science (https://www.webofscience.com), and China National Knowledge Infrastructure (http://www.cnki.net). All data used for the analyses can be downloaded from the Figshare repository (https://doi.org/10.6084/m9.figshare.24231622), and can also be downloaded at resistancebank.org (https://resistancebank.org).

Research involving human participants, their data, or biological material

Policy information about studies with human participants or human data. See also policy information about sex, gender (identity/presentation), and sexual orientation and race, ethnicity and racism.

Reporting on sex and gender	N/A
Reporting on race, ethnicity, or other socially relevant groupings	N/A
Population characteristics	N/A
Recruitment	N/A
Ethics oversight	N/A

Note that full information on the approval of the study protocol must also be provided in the manuscript.

Field-specific reporting

Please select the one below that is the best fit for your research. If you are not sure, read the appropriate sections before making your selection.

☐ Life sciences ☐ Behavioural & social sciences ☒ Ecological, evolutionary & environmental sciences

For a reference copy of the document with all sections, see nature.com/documents/nr-reporting-summary-flat.pdf

Ecological, evolutionary & environmental sciences study design

All studies must disclose on these points even when the disclosure is negative.

Study description	We extracted point-prevalence surveys on antimicrobial resistance in E. coli and Salmonella isolated from food animals in low- and middle-income countries. We used geospatial models to map resistance prevalence for seven antimicrobials. We then developed an approach to map the antimicrobial with the highest probability of its resistance prevalence exceeding critical levels in the future. Because what we presented here were observational studies without experimental designs, we did not have experimental units and replicates.
Research sample	Indicator bacteria (E. coli and non-typhoidal Salmonella) isolated from a total of 351,757 field samples of food animals in 1,088 point-prevalence surveys. These two types of bacteria were the most commonly tested bacteria in food animals, and were therefore chosen in this study. The collected data were meant to represent food animals in low- and middle-income countries, which were around 21,726,693,000 chickens, 1,220,938,612 cattle, and 1,175,643,333 pigs in 2015 (source: FAOSTAT).<br>We used literature review from Van Boeckel et al. 2019, Zhao et al. 2020, and also conducted additional literature review in this study (see Supplementary Methods for details).
Sampling strategy	The point-prevalence surveys used in this analysis were previously extracted through multiple literature reviews of publications between 2000 and 2019 in low- and middle-income countries, from four major search engines - PubMed, Scopus, ISI Web of Science, as well as China's National Knowledge Infrastructure. We also included an additional 73 publications in this study. There was no predetermination of sample size - we extracted all publications that met the inclusion criteria. The uncertainty of predictions based

on the available number of samples was quantified using maps of prediction uncertainty.

Data collection	We queried four major search engines following protocols described in Van Boeckel et al. 2019, and manually screened the titles and abstracts of the retrieved publications. We read the potentially relevant point-prevalence surveys in full, and extracted resistance prevalences and associated information from publications that met the inclusion criteria. The extraction was conducted by Cheng Zhao, Yu Wang, Joao Pires, Reshma Silvester, and Julia Song.
Timing and spatial scale	We conducted three rounds of literature search from four databases - PubMed, Scopus, ISI Web of Science, and China National Knowledge Infrastructure. The first round was conducted on 28.03.2019 from the first three aforementioned databases, and extracted data from all papers published between January 2000 and December 2018. The extracted data and details of literature review were published in Van Boeckel and Pires et al. 2019. The second round of literature search was conducted on 11.03.2020 from all four databases, and included surveys published between January 2000 and December 2019 exclusively for China. The extracted data and details of literature review were published in Zhao et al. 2020. The third round of literature search was conducted on 12.01.2022 from the first three aforementioned databases, and included all papers published between January 2019 and December 2019 in low- and middle-income countries apart from China. In summary, the literature search covered all point-prevalence surveys published between 2000 and 2019. The spatial scale covers all low- and middle-income countries.
Data exclusions	Exclusion criteria for the collected publications were pre-established, including: reviews; meta-analysis; surveys on diseased animals; experiments on antimicrobial effect of traditional Chinese medicine; manuscripts characterizing a defined set of strains not derived from point-prevalence surveys (strain surveys); surveys in which samples were pooled between host species, or resistance rates were pooled between bacteria; and studies without information on sampling locations. Out of 44,325 publications that we looked through, we excluded 43,237 publications based on these criteria and retained 1,088 publications for inclusion in our analysis (See Supplementary Table 1 for details).
Reproducibility	The reproducibility of our analyses was verified by re-running the codes and examining the outcomes.
Randomization	This does not apply to our study, because we did not conduct experiments that would require randomization.
Blinding	Blinding was not possible for our literature review, because we needed complete information from each paper in order to extract all relevant information.

Did the study involve field work? ☐ Yes ☒ No

Reporting for specific materials, systems and methods

We require information from authors about some types of materials, experimental systems and methods used in many studies. Here, indicate whether each material, system or method listed is relevant to your study. If you are not sure if a list item applies to your research, read the appropriate section before selecting a response.

Materials & experimental systems

n/a	Involved in the study	
☒	☐	Antibodies
☒	☐	Eukaryotic cell lines
☒	☐	Palaeontology and archaeology
☒	☐	Animals and other organisms
☒	☐	Clinical data
☒	☐	Dual use research of concern
☒	☐	Plants

Methods

n/a	Involved in the study	
☒	☐	ChIP-seq
☒	☐	Flow cytometry
☒	☐	MRI-based neuroimaging
